# Magnitude of tuberculosis treatment outcomes and associated factors in public health institutions of Arba Minch town, Southern Ethiopia: A multi-centered retrospective cross-sectional study

**DOI:** 10.1371/journal.pone.0338393

**Published:** 2025-12-08

**Authors:** Behailu Asmamaw, Temesgen Tamiru Tadese, Rediet Yifru Mamo, Berhanu Amare Taye, Abrham Workineh Azale, Yohannes Chemere Wondmeneh, Dagmawi Nega Shibeshi, Eunice Borkor Bortequaye, Temesgen Teklu Ziza, Temesgen Esayas Bolliso, Tiwabwork Tekalign, Awoke Guadie

**Affiliations:** 1 School of Medicine, College of Medicine and Health Sciences, Arba Minch University, Arba Minch, Ethiopia; 2 School of Medicine, College of Medicine and Health Sciences, Addis Ababa University, Addis Ababa, Ethiopia; 3 School of Medicine, College of Medicine and Health Sciences, University of Gondar, Gondar, Ethiopia; 4 Department of Internal Medicine, Yekatit 12 Medical College, Addis Ababa, Ethiopia; 5 School of Medicine, University for Developmental Studies, Tamale, Ghana; 6 Rollins School of Public Health, Emory University, Atlanta, Georgia, United States of America; 7 School of Medicine, College of Medicine and Health Science, Hawassa University, Awasa, Ethiopia; 8 School of Nursing, College of Medicine and Health Sciences, Arba Minch University, Arba Minch, Ethiopia; 9 Department of Biology, College of Natural Sciences, Arba Minch University, Arba Minch, Ethiopia; The University of Georgia, UNITED STATES OF AMERICA

## Abstract

**Background:**

In Ethiopia, the incidence rate of tuberculosis (TB) has been steadily increasing, from 119 per 100,000 in 2021–126 per 100,000 in 2022 and 146 per 100,000 in 2023. Moreover, TB remains the second leading cause of death after malaria, and the third leading cause of hospital admissions. So, the aim of this study is to determine the treatment outcome and associated factors in the public health institutions of Arba Minch town.

**Methods:**

A multi-centered retrospective cross-sectional study was conducted involving 609 tuberculosis patients admitted from September 2021-August 2024 at public health institutions in Arba Minch town. A structured data extraction form was used by trained research assistants to collect the data from TB patient registration record books. Variables with a p-value <0.25 in binary logistic regression were further analyzed using multivariable logistic regression. Statistically significant factors were considered those with a p-value <0.05.

**Result:**

The majority (53.7%) of the participants were aged between 21 and 40 years. Most participants were diagnosed with pulmonary tuberculosis (71.8%) and found to be new (92.9%) patients. According to this study, the magnitude of successful treatment outcomes was found to be 86.9%. In the multivariate logistic regression, being unmarried (p = 0.023), educational level (p = 0.028), and having extra-pulmonary TB (p = 0.017) have been found significantly associated with successful treatment outcome.

**Conclusion:**

The study indicates a relatively positive rate of successful treatment outcomes for TB. Although the treatment outcome results are positive, targeted interventions are needed for individuals who are married, have a low educational status, and have been diagnosed with pulmonary tuberculosis.

## Introduction

Tuberculosis (TB) has continued to be one of the major public concerns worldwide, being the most fatal infectious disease, killing nearly 1.25 million people in 2023. According to the 2024 WHO TB report, it is estimated that nearly 10.8 million people are affected with TB, among these people living in the 30 nations labeled as high-burden countries account for nearly 90% of the cases [1]. It is a disease that disproportionately affects people living in resource-limited settings [2]. Low- and middle-income countries, including Sub-Saharan Africa, account for 94% of TB-related illnesses and deaths [3]. Tuberculosis is both a preventable and curable disease that is caused by the intracellular pathogen, *Mycobacterium tuberculosis.* Although TB mainly affects the lungs (pulmonary TB), it can also involve any other organ in the body like lymph nodes, pleura, intestine, bone and soft tissues, and central nervous system which are collectively termed as extra-pulmonary TB (EPTB) [4].

Ethiopia, a country in Eastern Africa, is among the 30 high-burden countries for TB and TB/HIV, with an estimated incidence rate of 146 per 100,000 population (188,000 cases) and 25,000 TB-related deaths in HIV-negative individuals (19 per 100,000). The incidence rate has been steadily increasing from 119 per 100,000 in 2021–126 per 100,000 in 2022 and 146 per 100,000 in 2023 [5]. Moreover, TB remains the second leading cause of death after malaria, and the third leading cause of hospital admissions following deliveries and malaria, according to the Ethiopian Federal Ministry of Health (FMOH) [6]. These trends highlight TB as a serious public health concern that requires urgent intervention.

The treatment success rate (TSR) for TB varies across African nations, ranging from 53% in Nigeria to 92% in Ethiopia (2010–2020) [7]*.* According to the Global TB Report 2024, the Ethiopian (national) TSR among newly diagnosed TB patients is 86%, and 77% for patients with HIV co-infection, which is lower than the WHO African and global averages and the target set by the end TB strategy [1]. The TSR also varies significantly among different regions of Ethiopia, ranging from 82.5% in western Ethiopia to 92% in Eastern Ethiopia [8–12]. Previous studies showed that being male, having HIV co-infection, age > 45 years, rural residence, underweight, retreatment, smear-negative cases, patients with no adherence support, limited access to transport, distance from home to a treatment center, and low income were factors associated with poor treatment outcomes [8–13].

According to the end TB strategy, set a milestone for the year 2025, reaching the target of 90% TSR needs intensive research efforts and rigorous monitoring and evaluation of outcomes [14]. While Ethiopia has made significant progress in TB control, there is limited evidence on region-specific treatment outcomes and associated factors. Regular monitoring of outcomes and predicting factors is of paramount significance regarding addressing specific challenges faced by different population segments and making tailored decisions. There is limited data on TB treatment outcomes and associated factors in Southern Ethiopia, specifically in the Gamo zone, Arba Minch town. This study aims to fill this gap by identifying key determinants of treatment outcomes, which can inform targeted interventions to improve TB care and reduce disease burden.

Therefore, the objective of this study is to assess the treatment outcomes and associated factors among TB patients in public health institutions of Arba Minch town, Southern Ethiopia.

## Methods

### Description of the study area

This facility-based retrospective study included TB patients treated between September 2021 and August 2024 to assess the magnitude of treatment outcomes and associated factors among patients with confirmed TB in Arba Minch town, Southern Ethiopia. Gamo zone is one of the six zones and five special woredas (districts) which are found in the Southern Ethiopia Regional State, Arba Minch being its major town. Arba Minch town is located 505 km south of Addis Ababa, the capital city of Ethiopia, and has an estimated population size of more than 200,000 people (with an estimated population of male 101,000 and female 100,000) [15]. In Arba Minch town, there are two hospitals (Dilfana Primary Hospital and Arba Minch General Hospital) and one health center (Secha Health Center).

### Study population and criteria for inclusion and exclusion

All TB patients who were diagnosed with and treated for TB at Dilfana Primary Hospital, Arba Minch General Hospital, and Secha Health Center were the source population.

### Eligibility criteria

#### Inclusion criteria.

The data of all patients diagnosed with TB and who were initiated on anti-TB treatment in public health institutions of Arba Minch town during the study period were included in the study. Patients, who had full information in the unit TB register including clearly documented treatment outcomes, were considered eligible.

#### Exclusion criteria.

Patients who were referred to other health facilities and/or had incomplete information were excluded from the study. Records were considered incomplete if they missed data on essential variables such as age, sex, or treatment outcomes (cured, treatment completed, treatment failure, loss to follow-up, death, or not evaluated).

### Study design and sampling procedure

**Fig 1:**
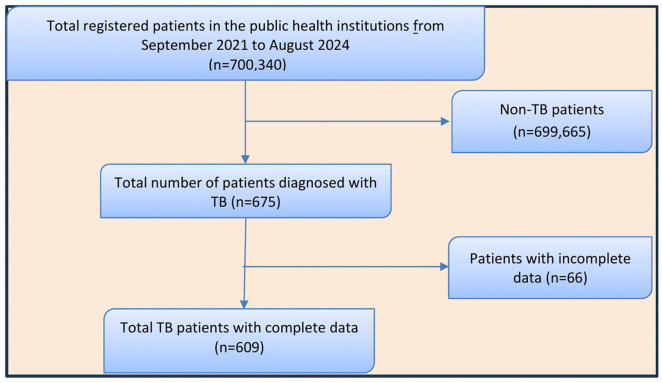
Sampling procedure for the selection of tuberculosis patients with complete data.

This multi-centered, facility based retrospective study was conducted in public health facilities of Arba Minch town, Southern Ethiopia. Between September 2021 and August 2024, a total of 700,340 patients were seen across these public health institutions for various health services. Of these, 675 patients were diagnosed with all forms of tuberculosis (TB) and initiated on anti-TB treatment at the three facilities (Fig 1). Among them, 609 patients fulfilled the eligibility criteria and were included in the study (Fig 1). To maximize representativeness, eliminate sampling error, and enhance the reproducibility of the findings, the study employed a census of all eligible TB patients during the study period.

### Data collection and diagnosis

Data on treatment outcomes and independent variables were collected from TB clinic-based unit TB registers and patient medical charts. Data for this retrospective study were accessed from unit TB registers and medical charts between January 20 and February 20, 2025, for research purposes. The data collected included demographic information such as gender, age, weight, and body mass index (BMI). The study also collected many clinical characteristics such as the type of TB, TB registration category, GeneXpert *Mycobacterium tuberculosis*/rifampicin resistance (MTB/RIF) assay diagnosis results, HIV status, initiation of antiretroviral therapy (ART), and indications of presumptive drug resistance (PDR). During data collection experienced staff working in the TB treatment units were involved. They were trained and collected under the strict supervision of the principal investigator. The data were collected using a pretested structured data extraction questionnaire that included the above-mentioned information.

In these institutions, TB-suspected patients were first identified by examining the signs and symptoms, prior history of TB, and chest x-ray results. In addition, sputum samples were sent for a molecular drug susceptibility test to confirm the diagnosis and make sure they don’t have a drug resistance pattern. The final diagnosis of TB was made according to the recommendations of the Ministry of Health (MoH) of Ethiopia [6]. Treatment was given based on national guidelines for TB treatment. This included a full course of anti-TB therapy for a total of six months. Two-month intensive phase with four major drugs (isoniazid, rifampicin, pyrazinamide, and ethambutol), and an additional four-month continuation phase with rifampicin and isoniazid. Outcomes were labeled successful (cure/complete) and unsuccessful (death, lost-to-follow-up, failure, not evaluated) [6].

### Operational definition

**Cured:** A TB patient who started treatment with bacteriologically verified TB and finished the treatment as advised by national policy with evidence of a bacteriological response but no signs of failure [16].

**Treatment completed:** A TB patient who followed the national policy suggested a course of therapy but whose results did not fulfill the criteria for cure or treatment failure [16].

**Treatment failure:** A TB patient whose treatment plan required being stopped or permanently switched to another treatment plan [16].

**Died:** A TB patient who passed away for whatever reason, either before beginning treatment or while receiving it [16].

**Lost to follow-up:** A TB patient who has not started therapy or whose regimen has been stopped for eight or more weeks in a row after starting treatment at least four weeks previously [16].

**Not evaluated:** A TB patient for whom no treatment result has been established. This applies to situations where the reporting unit is unsure of the treatment outcome and situations where the case has been transferred to another treatment facility [16].

**New patients:** Patients with TB who have never undergone TB therapy or who have just started receiving anti-TB drugs. New patients may have positive or negative bacteriology and may have disease in any area of the body [16].

**Previously treated:** A TB patient may have the disease at any anatomical site, positive or negative bacteriology, and have taken anti-TB medications for at least one month in the past [16].

**Successful treatment outcome:** If TB patients finished therapy with symptom clearance or were cured (i.e., had a negative smear microscopy at the end of treatment and on at least one prior follow-up test) [16].

**Unsuccessful treatment outcome:** If TB patients had treatment but had treatment failure (i.e., were still smear-positive after five months), were lost to follow-up (i.e., patients who stopped taking their medication for two or more months consecutively after registering), or died [17].

### Data quality control

To minimize potential biases, a pre-test was conducted with 5% of the final sample size before the main data collection, and the checklist was revised based on the findings to ensure completeness and clarity. The pre-tested, structured questionnaire, prepared in English, was checked for consistency after the pretest.

The data collectors and a supervisor were trained for two days on the purpose of the study and the data collection procedures. All finalized data were reviewed for completeness and clarity by the principal investigator before and during data management, storage, and analysis.

### Data processing and analysis

Data collected for this study were managed using EpiData version 3.1 software. The data were thoroughly checked for completeness, coded, and entered into the software. The data was then exported and analyzed using SPSS version 26.

Due to the dichotomous nature of the outcome variable (successful vs. unsuccessful treatment outcomes), logistic regression analysis was employed. This model was specifically chosen for its ability to estimate the odds of an event occurring while effectively adjusting for multiple covariates. Initially, a binary logistic regression analysis was performed to identify variables associated with the treatment outcome at a crude level. Any variables with a p-value of <0.25 in the binary analysis were considered a potential candidate for inclusion in the final (i.e., multivariable logistic regression) model to control for potential confounding and to identify independent predictors of treatment outcome. Statistical significance in the final model was established at a p-value of ≤0.05.

Finally, model fitness was assessed using the Hosmer–Lemeshow goodness-of-fit test, and multicollinearity among independent variables was checked to ensure the reliability of the final regression model.

### Ethical approval and consent to participants

The ethical approval to conduct this study was obtained from the Arba Minch University Ethical Review Board (IRB/23304/25), and a cooperation letter was given to the facilities. This study had no contact with patients, and the information obtained regarding patients was made anonymous.

## Result

### Socio-demographic characteristics of the participants

A total of 609 participants enrolled in this study were systematically analyzed from the total (700,340) population attending in the three health centers from September 2021 to August 2024. The majority of the participants were aged between 21 and 40 years, with 327 (53.7%) individuals falling within this age range. Additionally, 380 (62.4%) participants were male. More than half (53.9%) of the participants, were married, and over two-thirds (77.7%) participants had received formal education. Furthermore, 326 (54.6%) participants were classified as underweight (Table 1).

**Table 1:** Socio-demographic characteristics of study participants.

Variables	Characteristics	Frequency	%
Age (year)	<20	129	21.2
	21-40	327	53.7
	41-60	111	18.2
	>60	42	6.9
Gender	Male	380	62.4
	Female	229	37.6
Marital status	Unmarried	281	46.1
	Married	328	53.9
Educational level	Informal education	136	22.3
	Formal education	473	77.7
Body mass index	Under weight	326	54.6
	Normal weight	251	42.1
	Overweight	19	3.1

### Clinical and behavioral related characteristics

More than half (52.1%) of the participants were reported no history of alcohol consumption, while a greater (76.5%) proportion did not have a history of cigarette smoking. Most (71.8%) participants were diagnosed with pulmonary TB and greater than 90% were found new patients (Table 2). All participants were offered an HIV test; among them, the majority (78.2%) of the results were found non-reactive. Of those who tested reactive (21.8%), the vast majority (98.4%) were already began antiretroviral therapy. Additionally, 607 (99.6%) of the participants did not show signs of PDR, and 413 (67.8%) did not undergo the GeneXpert MTB/RIF test. 17 (2.8%) Patients who couldn’t expectorate sputum, and debilitated HIV/ AIDS patients with low cluster of differentiation (CD4) count were evaluated with urine Lateral Flow-Lipoarabinomanan test (LF-LAM). Furthermore, a significant number (n = 443) of participants had a positive smear test result (Table 2).

**Table 2:** Clinical and behavioral characteristics of study participants.

Variables	Characteristics	Frequency	%
History of alcohol	Yes	292	47.9
	No	317	52.1
History of smoking	Yes	143	23.5
	No	466	76.5
Type of TB	Pulmonary TB	437	71.8
	Extra pulmonary TB	156	25.6
	Unknown	16	2.6
Category of TB	New	566	92.9
	Repeat	37	6.1
	Transfer in	6	1.0
HIV comorbidity	Reactive	133	21.8
	Non-reactive	476	78.2
ART initiation	Yes	131	98.5
	No	2	1.5
PDR	Yes	2	0.4
	No	607	99.6
GeneXpert MTB/RIF assay performed	Yes	179	29.4
	No	413	67.8
	LF-LAM	17	2.8
Smear examination	Positive	443	72.7
	Negative	162	26.6
	Not done	4	0.7

TB = Tuberculosis, HIV = Human immunodeficiency Virus, ART = Antiretroviral therapy, PDR = Presumptive drug resistance, LF-LAM = Later flow Lipoarabinomannan.

### TB treatment outcomes

**Fig 2:**
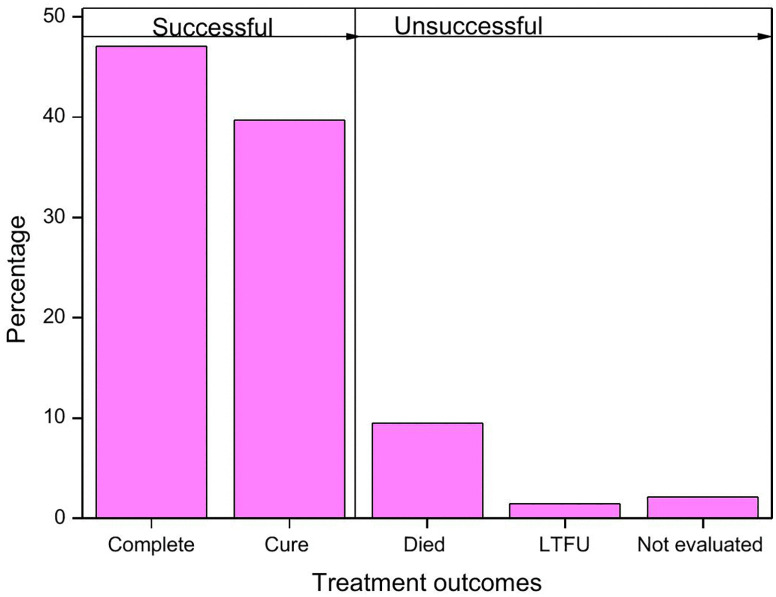
Treatment outcome of TB patients treated at public health institutions of Arba Minch town.

According to this study, out of the 609 patients, 529(86.9%) TB patients had a successful treatment outcome. The cure rate for TB patients who received treatment was 39.1%, and the treatment completion rate was 47.15%. Whereas 58 patients (9.5%) died, 9 patients (1.5%) defaulted and the outcome of 13 patients (2.1%) was declared not evaluated making the total unsuccessful treatment outcome 13.1% (Fig 2).

### Factors associated with successful TB treatment outcome

Seven variables in binary logistic regression with a p-value of less than 0.25 became candidates for multiple logistic regressions (Table 3). In multiple logistic regressions, three variables were significantly associated (Table 3) with treatment outcome of TB with p-value <0.05. These are educational status (p = 0.028), being unmarried (p = 0.023), and having extra-pulmonary TB (p = 0.017) (Table 3).

**Table 3:** Factors associated with tuberculosis treatment outcome in public health institutions of Arba Minch town (n = 609).

Variables	Category	TB-TO		COR (95% CI)^a^	P-value	AOR (95% CI)^b^
		Successful	Unsuccessful			
Educational Status	Informal education	100	36	1		1
	Formal education	380	93	0.68(0.43-1.05)	0.028	1.67(1.05-2.66) ***
Marital status	Unmarried	210	71	1.57(1.06- 2.32)	0.023	1.64(1.05-2.47) ***
	Married	270	58	1		1
Alcohol history	Yes	237	55	1.31(0.88-1.94)	0.174	1.08(0.70-1.67)
	No	243	74	1		1
Smoking history	Yes	361	105	1		1
	No	119	24	0.69(0.42-1.13)	0.143	1.40(0.81-2.42)
Type of TB	Pulmonary	335	102	1		1
	Extra pulmonary	135	21	3.85(1.26-11.77)	0.017	3.80(1.23-11.7) ***
	Unknown	10	6	1.97(0.69-5.55)	0.199	2.00(0.69-5.74)
HIV-Coin	Yes	113	20	1.67(0.99-2.82)	0.052	0.62(0.36-1.06)
	No	367	109	1		1
ART-Init	Yes	111	20	1.63(0.97-2.76)		1.89(0.14-24.5)
	No	369	109	1		1

TB-TO=TB treatment outcome, COR = Crude Odd Ratio, AOR = Adjusted Odds Ratio, HIV-Coin = Human Immunodeficiency Virus Co-infection, ART-Init = Antiretroviral initiation, *** = p < 0.05. Values in a column indicated in letters “a” and “b” show the bivariate and multivariate logistic regression results respectively.

Those study participant who had formal education were 2 times more likely to have successful treatment outcome (AOR = 1.67; 95% CI = 1.05–2.66; p = 0.028) than their counterparts. Those participants who were unmarried during the time of treatment were 2 times more likely to have successful treatment outcome (AOR = 1.64; 95% CI = 1.05–2.47; p = 0.023) than married participants and participants with EPTB were almost 4 times more likely to have successful treatment outcome (AOR = 3.8; 95% CI = 1.23–11.7; p = 0.017) than participants with pulmonary TB.

## Discussion

Globally, TB is a significant public health issue, with one of the highest morbidity and mortality rates. It is estimated that millions of new cases arise each year, particularly in regions with limited healthcare resources, where factors like poverty, malnutrition, and the prevalence of HIV/AIDS further exacerbate the situation [18,19]. In this study, treatment outcome and factors associated with TB were assessed. The magnitude of successful treatment outcomes among TB patients was found to be 86.9%, which is a bit lower than the world (88%) and African (88%) average TB treatment success rate for new and relapse cases reported in 2024 [1]. However, this result is nearly similar to studies conducted in Gibe Woreda, Southern Ethiopia (85.5%) [12] and Debre Tabor Town, Northwestern Ethiopia (87.1%) [20]. Such consistency in outcomes could suggest comparable healthcare practices or disease management strategies in these regions (Table 4).

**Table 4:** Comparing TB treatment outcome and associated factors in prior studies.

Study area	Sample size (number)	Methodology	TSR (%)	Associated factors	Reference
Arba Minch, Ethiopia	609	Retrospective	86.9	Formal education, unmarried, and EPTB (+)	This study
Gondar, Ethiopia	1584	Retrospective cross-sectional	60.1	Age 30–39.9, smear negative, and being EPTB, retreatment, HIV positive (-)	[21]
Woldia, Ethiopia	270	Cross-sectional	80.7	Older age, female sex, year of registration in 2015, and being HIV positive (-)	[22]
Afar, Ethiopia	380	Retrospective	81.8	Older age, HIV Reactive status (-); while (four-week attendance, sputum follow-up test, and being female (+)	[23]
Wolaita, Ethiopia	232	Retro-cohort	82.5	No statistically significant associated factor	[10]
Gibe, Ethiopia	400	Cross-sectional	85.5	Age greater than 45, male sex, travel distance over 10 km, lack of family support, bedridden functional status (-)	[12]
Debretabor, Ethiopia	303	Retrospective	87.1	Female sex, rural residence, smear negative at 2^nd^ month of treatment (-)	[20]
Jimma, Ethiopia	1249	Retrospective	88.2	Smear-negative, Extra-pulmonary TB, unknown HIV status (-)	[24]
Harare, Ethiopia	1236	Retrospective	92.5	Female, HIV negative, weight 20–29 kg (+); while Age > 54 (-)	[8]
Enfranz, Ethiopia	417	Retrospective	94.8	No statistically significant associated factor	[25]
Easter Cape, South Africa	427	Ambidirectional	65.8	HIV co-infection, history of smoking (-)	[26]
Ashanti region, Ghana	891	Retrospective	68.4	Retreatment (-); while Age less than 20 or age range b/n 51–60, pre-treatment weight > 35 kg (+)	[20]
Anambra & Oyo, Nigeria	1281	Cross-sectional	75.8	Geographical location, facility tier/type (-)	[27]
Mogadishu, Somalia	385	Cross-sectional	81.5	Married, educated, HIV-negative, new treatment, knowledge of TB (+)	[28]
Hunan province, China	308,860	Retro-cohort	98.6	Age, male, being severally ill, TB treatment history, year of diagnosis (-)	[29]
Ahmedabad, India	8301	Retro-cohort	87.9	Age 45–54 (-)	[30]
Ethiopia*	NS	Survey report	86.0	Age, gender, HIV, under-nutrition, diabetes mellitus, alcohol, drug resistance, smoking, poverty (-)	[1]
Africa*	NS	Survey report	88.0	Age, gender, HIV, under-nutrition, diabetes mellitus, alcohol, drug resistance, smoking, poverty (-)	[1]
World*	NS	Survey report	88.0	Age, gender, HIV, under-nutrition, diabetes mellitus, alcohol, drug resistance, smoking, poverty (-)	[1]

(+)= Significantly associated with successful treatment outcomes, (-)=Significantly associated with unfavorable treatment outcomes, EPTB = Extra-pulmonary tuberculosis, TSR: = Treatment success rate, Retro-cohort = Retrospective cohort, * = Average report in 2024, NS = Not stated, Prosp-cohort = Prospective cohort.

In contrast to our finding, studies from various locations have reported significantly lower TB treatment success rates in Ethiopia and other countries in Africa. For example, a study in Ghana (five directly observed therapy short-course (DOTS) centres at the Atwima Nwabiagya district) found a lower TB treatment success rate (68.5%) [31], while in South Africa (Eastern Cape hospitals) recorded an even lower (65.8%) rate of treatment success [26]. Similarly, studies in two Nigeria states (Anambra and Oyo) and Somalia (across seven public TB management units in Mogadishu) demonstrated a lower TB treatment success rate of 75.8% [27] and 81.8% [28], respectively. Lower TB treatment success rates were also observed across various regions in Ethiopia, highlighting significant internal differences that may reflect localized challenges in healthcare delivery. Specifically, TB treatment success rates reported in the Afar region (81.8%) [32], Gonder (60.1%) [21], Wolayta (82.5%) [10], and Woldia (80.7%) [22] (Table 4). These regional variations may be attributed to several factors, including differences in study periods and sample sizes. The discrepancies also highlight how regional healthcare systems and patient demographics ultimately influence adherence and treatment completion. A comprehensive understanding of these discrepancies is essential for designing effective TB control strategies across the country.

On the other hand, the current study TB treatment success rate was lower than those reported in Harar, Eastern Ethiopia (92.5%) [8], Jimma, Southwestern Ethiopia (88.3%) [24], and Enfraz Health Center, Northwestern Ethiopia (94.8%) [25] (Table 4). The higher success rates in these studies may be attributed to different methodologies, particularly the use of larger sample sizes, which typically enhance the reliability of findings. Moreover, the types of healthcare facilities and their level may significantly influence treatment outcomes. Factors such as the demographic characteristics of the patient population and the degree of urbanization in the settings could also play a crucial role in affecting these outcomes. In addition, our treatment success rate is lower than that reported in Hunan Province, China (98.6%) [29] as well as the national average of 95% in 2022 [1]. This difference may be attributed to China’s strong universal health coverage, substantial TB financing, and the consistent application of DOTS through its National TB Control Program. Similarly, in India 87.9% of TB treatment success rate were reported in 2023 [30], supported by its Revised National TB Control Program, which emphasizes early case detection, patient-centered treatment, and enhanced monitoring systems.

Multivariate logistic regression model analysis showed that participants who were unmarried during the time of treatment had 64% higher odds of having a successful treatment outcome (AOR = 1.64; 95% CI = 1.05–2.47; p = 0.017) compared to those who were married. This finding contrasts with previous studies, which demonstrated unmarried individuals had a lower social support system [10,22,28]. The most likely explanation could be due to married individuals in the Gamo zone may face competing family and household responsibilities, such as caregiving, income-generating activities, and maintaining household obligations, which could interfere with adherence and clinic attendance. Indeed, other studies in Southern Ethiopia found that being married was associated with lower odds of successful TB treatment outcome [33]. A study conducted within Gamo Gofa zone (a similar study area for our study) also highlighted that family dynamics, such as a patient’s failure to disclose their TB status to household members, were strongly associated with non-adherence [34]. Based on our finding and these evidences [33,34], the burden of marital and household commitments are critical contributing factors to suboptimal treatment success in this specific context, suggesting the vital need for future research to deeply explore and mitigate these underlying mechanisms. Our study is deficient in addressing how factors such as socioeconomic status, cultural norms and stigma, and household dynamics affect the treatment outcome among married patients. Future studies should explore a longitudinal or mixed-methods design to explore how the interplay of those factors and marital status affect the treatment of TB.

The educational status of the participants showed statistically significant association with treatment outcomes, particularly those who attended formal education were almost twice as likely to achieve a successful treatment outcome (AOR = 1.67; 95% CI = 1.05–2.66; p = 0.028) compared to those without formal education. This finding is consistent with a study conducted in Somalia [28]. Various pieces of evidence indicate that individuals with higher levels of education tend to have better health literacy, medication adherence, improved health-seeking behavior, and the overall socioeconomic advantages that education provides [35–37].

Participants with EPTB were almost 4 times more likely to have successful treatment outcome (AOR = 3.8; 95% CI = 1.23–11.7, p = 0.023) than participants with pulmonary TB. This finding contrasts with most of the previous studies [8,10,21,22,24,28]. The possible explanation might be some of the EPTB infections such as infections involving lymph nodes may have a more localized and less aggressive presentation compared to pulmonary TB, EPTB patients might have stronger immune reactivity when the infection is confined to specific sites, and patients with pulmonary TB might face additional challenges due to respiratory complications such as respiratory failure, which can complicate treatment and lead to poorer outcomes. Previous studies demonstrated variable results on the treatment success rate of EPTB, which is dependent on different factors such as location of the infection, HIV co-morbidity, and age [38,39]. Those patients presenting with EPTB, particularly involving the pleura, lymph nodes, and abdomen, had better outcomes. However, those infections involving the central nervous system carry poor outcomes [38]. Since our data couldn’t identify the specific types of EPTB, future studies needed that focus on incorporating data on specific types of EPTB in their studies.

Although many studies [1,8,22] stated that HIV positive, advanced age, male sex, and malnutrition are commonly associated with unfavorable TB treatment outcomes, our study did not find significant associations with these factors. The finding that HIV positive status was not statistically associated with poor TB treatment outcomes in our study, contrary to global trends, likely originates from the effectiveness of the local integrated TB-HIV collaborative services. The most probable primary reason is the high (98.5%) ART coverage and timely initiation (Table 2) among TB-HIV co-infected patients. Antiretroviral therapy rapidly suppresses the HIV virus, leading to immune reconstitution, which allows the patient’s body to respond effectively to the anti-TB drugs, mitigating the risk of death and treatment failure typically seen in immunocompromised individuals [1].

The prominent consumption of low-fat staple foods in the study area like *Enset* (including its products: *Kocho*, *Bulla*, and *Godere*), may directly contribute to lower population BMI values. Since BMI is a simple measure that does not differentiate between lean muscle mass and fat stores, a low BMI in this context may primarily reflect limited dietary fat intake rather than severe protein-energy malnutrition. Consequently, BMI may be an inadequate indicator of specific nutritional deficits (e.g., in essential fatty acids or fat-soluble vitamins) crucial for immune function and recovery. This limitation likely accounts for the observed lack of association between BMI and the measured TB treatment outcomes in this population.

To figure out the current study TB treatment outcome from other previous TB studies, a comparative Table (Table 4) is prepared.

Our study has limitations. It is based on retrospective analysis of medical records, which were not designed for research, which lacked data on factors such as level of income, social and cultural roles of married individuals, accessibility to health care, specific types of EPTB and longitudinal insights regarding the challenges faced by TB patients. Despite these limitations, our study provides information about the treatment outcome and associated factors among TB patients in Arba Minch town where data has not been previously published.

## Conclusion

In this study, the overall TB treatment success rate was found to be 86.9%, which is significantly associations with being unmarried, attending formal education, and being diagnosed with EPTB. This finding is below the target set by the WHO end TB strategy, which aims to achieve a TSR of 90% by 2030. This indicates that attention should be given to patients who are married, individuals with low educational levels, and patients diagnosed with pulmonary TB in order to improve the overall outcome of TB treatment in the Gamo zone. While the study benefits from being multi-centered and large sample size, future studies should focus on longitudinal studies to identify other potential socioeconomic and cultural factors that could affect the treatment outcomes of TB patients.

## References

[1] World Health Organization. Global tuberculosis report 2024. 1st edition. Geneva: World Health Organization; 2024.

[2] Litvinjenko S, Magwood O, Wu S, Wei X. Burden of tuberculosis among vulnerable populations worldwide: an overview of systematic reviews. Lancet Infect Dis. 2023;23(12):1395–407. doi: 10.1016/S1473-3099(23)00372-9 37696278 PMC10665202

[3] Jama SS, Abdi MM. Tuberculosis treatment outcomes and associated factors among patients treated at Bosaso TB Hospital, Bosaso, Somalia: A five-year retrospective study. PLoS One. 2025;20(1):e0314693. doi: 10.1371/journal.pone.0314693 39854532 PMC11761147

[4] Federal Ministry of Health (FMOH), Ethiopia. Guidelines for clinical and programmatic management of TB, TB/HIV, DR-TB and leprosy in Ethiopia. Addis Ababa: FMOH; 2021.

[5] World Health Organization. Tuberculosis profile: Ethiopia. Geneva: WHO; 2024.Available from: https://worldhealthorg.shinyapps.io/tb_profiles/

[6] FMOH, Ethiopia. Leprosy and TB/HIV Prevention and Control Programme Manual. Addis Ababa: FMOH; 2008.

[7] Teferi MY, El-Khatib Z, Boltena MT, Andualem AT, Asamoah BO, Biru M, et al. Tuberculosis Treatment Outcome and Predictors in Africa: A Systematic Review and Meta-Analysis. Int J Environ Res Public Health. 2021;18(20):10678. doi: 10.3390/ijerph182010678 34682420 PMC8536006

[8] Tola A, Minshore KM, Ayele Y, Mekuria AN. Tuberculosis Treatment Outcomes and Associated Factors among TB Patients Attending Public Hospitals in Harar Town, Eastern Ethiopia: A Five-Year Retrospective Study. Tuberc Res Treat. 2019;2019:1503219. doi: 10.1155/2019/1503219 31057963 PMC6463571

[9] Berhan A, Almaw A, Solomon Y, Legese B, Damtie S, Erkihun M, et al. Tuberculosis Treatment Outcome and Associated Factors Among Tuberculosis Patients Linked to Tuberculosis Treatment Clinics in Ethiopia, 2023: A Multi-Center Retrospective Study. Infect Drug Resist. 2023;16:3367–78. doi: 10.2147/IDR.S413272 37274357 PMC10239251

[10] Teferi MY, Didana LD, Hailu T, Woldesenbet SG, Bekele S, Mihret A. Tuberculosis treatment outcome and associated factors among tuberculosis patients at Wolayta Sodo Teaching and Referral Hospital, Southern Ethiopia: a retrospective study. J Public Health Res. 2021;10(3):2046. doi: 10.4081/jphr.2021.2046 33882659 PMC8419628

[11] Kassa JI, Dedefo MG, Korsa AT, Dibessa TT. Factors Affecting Treatment Outcome of Tuberculosis among Tuberculosis Patients in West Ethiopia. J Bioanal Biomed. 2018;10(1). doi: 10.4172/1948-593x.1000200

[12] Mengesha MM, Gebremichael MA, Watumo D, Hallström IK, Jerene D. Poor adult tuberculosis treatment outcome and associated factors in Gibe Woreda, Southern Ethiopia: An institution-based cross-sectional study. PLOS Glob Public Health. 2022;2(3):e0000161. doi: 10.1371/journal.pgph.0000161 36962264 PMC10021194

[13] Berihe Hiluf S, Abera A, Bahiru M, Kassie B. Determinants of unsuccessful tuberculosis treatment outcome in Southwest Ethiopia regional state public hospitals, 2022: a multi-center case control study. Front Public Health. 2024;12:1406211. doi: 10.3389/fpubh.2024.1406211 39502821 PMC11534863

[14] Uplekar M, Weil D, Lonnroth K, Jaramillo E, Lienhardt C, Dias HM, et al. WHO’s new end TB strategy. Lancet. 2015;385(9979):1799–801. doi: 10.1016/S0140-6736(15)60570-0 25814376

[15] Ethiopian Central Statistics Authority (CSA). Federal Democratic Republic of Ethiopia Central Statistical Agency. Population projection of Ethiopia for all regions. Available from: https://www.worldometers.info/world-population/ethiopia-population/#google_vignette

[16] World Health Organization. Treatment of tuberculosis: guidelines. 4th ed. Geneva: World Health Organization; 2010.

[17] Limenh LW, Kasahun AE, Sendekie AK, Seid AM, Mitku ML, Fenta ET, et al. Tuberculosis treatment outcomes and associated factors among tuberculosis patients treated at healthcare facilities of Motta Town, Northwest Ethiopia: a five-year retrospective study. Sci Rep. 2024;14(1):7695. doi: 10.1038/s41598-024-58080-0 38565912 PMC10987627

[18] Heaton B. Prevalence of tuberculosis in Sub-Saharan Africa. Ballard Brief. 2022;2022(1).

[19] Natarajan A, Beena PM, Devnikar AV, Mali S. A systemic review on tuberculosis. Indian J Tuberc. 2020;67(3):295–311. doi: 10.1016/j.ijtb.2020.02.005 32825856

[20] Melese A, Zeleke B, Ewnete B. Treatment Outcome and Associated Factors among Tuberculosis Patients in Debre Tabor, Northwestern Ethiopia: A Retrospective Study. Tuberc Res Treat. 2016;2016:1354356. doi: 10.1155/2016/1354356 27597896 PMC5002488

[21] Biruk M, Yimam B, Abrha H, Biruk S, Amdie FZ. Treatment Outcomes of Tuberculosis and Associated Factors in an Ethiopian University Hospital. Adv Public Health. 2016;2016:1–9. doi: 10.1155/2016/8504629

[22] Getie A, Alemnew B. Tuberculosis Treatment Outcomes and Associated Factors Among Patients Treated at Woldia General Hospital in Northeast Ethiopia: An Institution-Based Cross-Sectional Study. Infect Drug Resist. 2020;13:3423–9. doi: 10.2147/IDR.S275568 33116661 PMC7547773

[23] Zenebe T, Tefera E. Tuberculosis treatment outcome and associated factors among smear-positive pulmonary tuberculosis patients in Afar, Eastern Ethiopia: a retrospective study. Braz J Infect Dis. 2016;20(6):635–6. doi: 10.1016/j.bjid.2016.07.012 27553661 PMC9427637

[24] Abebe G, Bonsa Z, Kebede W. Treatment outcomes and associated factors in tuberculosis patients at Jimma University Medical Center: A 5-year retrospective study. Int J Mycobacteriol. 2019;8(1):35–41. doi: 10.4103/ijmy.ijmy_177_18 30860177

[25] Endris M, Moges F, Belyhun Y, Woldehana E, Esmael A, Unakal C. Treatment outcome of tuberculosis patients at enfraz health center, northwest ethiopia: a five-year retrospective study. Tuberc Res Treat. 2014;2014:726193. doi: 10.1155/2014/726193 24891948 PMC4027020

[26] Faye LM, Hosu MC, Iruedo J, Vasaikar S, Nokoyo KA, Tsuro U, et al. Treatment Outcomes and Associated Factors among Tuberculosis Patients from Selected Rural Eastern Cape Hospitals: An Ambidirectional Study. Trop Med Infect Dis. 2023;8(6):315. doi: 10.3390/tropicalmed8060315 37368733 PMC10300936

[27] Matthew Adebayo A, Olaiya Adeniyi B, Oluwasanu M, Hassan A, Ada Ajuwon G, Chidinma Ogbuji Q, et al. Tuberculosis treatment outcomes and associated factors in two states in Nigeria. Trop Med Int Health. 2020;25(10):1261–70. doi: 10.1111/tmi.13467 32677754

[28] Ali HM, Yusuf AM, Ahmed HM, Abdirahman MS. Factors associated with tuberculosis treatment outcomes among tuberculosis patients attending tuberculosis treatment centers in Mogadishu, Somalia, 2016–2017. Pan Afr Med J. 2018;30:49. 29610635 10.11604/pamj.2017.28.197.13439 PMC5878850

[29] Gilmour B, Xu Z, Bai L, Alene KA, Clements ACA. Risk factors associated with unsuccessful tuberculosis treatment outcomes in Hunan Province, China. Trop Med Int Health. 2022;27(3):290–9. doi: 10.1111/tmi.13720 35014123 PMC9305245

[30] Prajapati AC, Shah T, Panchal S, Joshi B, Shringarpure K, Jakasania A, et al. Treatment outcomes and associated factors among patients with drug-sensitive tuberculosis on daily fixed-dose combination drugs: A cohort study from Ahmedabad, India. J Family Med Prim Care. 2023;12(3):452–9. doi: 10.4103/jfmpc.jfmpc_1331_22 37122671 PMC10131945

[31] Agyare SA, Osei FA, Odoom SF, Mensah NK, Amanor E, Martyn-Dickens C, et al. Treatment Outcomes and Associated Factors in Tuberculosis Patients at Atwima Nwabiagya District, Ashanti Region, Ghana: A Ten-Year Retrospective Study. Tuberc Res Treat. 2021;2021:9952806. doi: 10.1155/2021/9952806 34336281 PMC8315879

[32] Panford V, Kumah E, Kokuro C, Adoma PO, Baidoo MA, Fusheini A, et al. Treatment outcomes and associated factors among patients with multidrug-resistant tuberculosis in Ashanti Region, Ghana: a retrospective, cross-sectional study. BMJ Open. 2022;12(7):e062857. doi: 10.1136/bmjopen-2022-062857 35790328 PMC9258480

[33] Agazhu HW, Assefa ZM, Beshir MT, Tadesse H, Mengstie AS. Treatment outcomes and associated factors among tuberculosis patients attending Gurage Zone Public Hospital, Southern Nations, Nationalities, and People’s Region, Ethiopia: an institution-based cross-sectional study. Front Med (Lausanne). 2023;10:1105911. doi: 10.3389/fmed.2023.1105911 37601784 PMC10436196

[34] Ajema D, Shibru T, Endalew T, Gebeyehu S. Level of and associated factors for non-adherence to anti-tuberculosis treatment among tuberculosis patients in Gamo Gofa zone, southern Ethiopia: cross-sectional study. BMC Public Health. 2020;20(1):1705. doi: 10.1186/s12889-020-09827-7 33187496 PMC7666453

[35] Zhu X, Wen M, He Y, Feng J, Xu X, Liu J. The Relationship Between Level of Education, Cognitive Function and Medication Adherence in Patients with Schizophrenia. Neuropsychiatr Dis Treat. 2023;19:2439–50. doi: 10.2147/NDT.S424694 38029047 PMC10657742

[36] Kilgore K, Pulungan Z, Teigland C, Parente A. The Impact of Demographic And Socio-Economic Factors on Medication Adherence. Value in Health. 2016;19(3):A289. doi: 10.1016/j.jval.2016.03.751

[37] Basha EA, Teshome DF. Medication adherence and its predictors among patients with chronic illness in nursing and midwifery settings. Nurs Midwifery Stud. 2018;7(2):45–51.

[38] Tahseen S, Khanzada FM, Baloch AQ, Abbas Q, Bhutto MM, Alizai AW, et al. Extrapulmonary tuberculosis in Pakistan- A nation-wide multicenter retrospective study. PLoS One. 2020;15(4):e0232134. doi: 10.1371/journal.pone.0232134 32343714 PMC7188211

[39] Ranzani OT, Rodrigues LC, Waldman EA, Carvalho CRR. Estimating the impact of tuberculosis anatomical classification on treatment outcomes: A patient and surveillance perspective analysis. PLoS One. 2017;12(11):e0187585. doi: 10.1371/journal.pone.0187585 29166408 PMC5699807

