# Supplementary material for: Magnitude of tuberculosis treatment outcomes and associated factors in public health institutions of Arba Minch town, Southern Ethiopia: A multi-centered retrospective cross-sectional study
Source: PLoS One. 2025 Dec 8;20(12):e0338393. doi: 10.1371/journal.pone.0338393 (PMC12685194; doi:10.1371/journal.pone.0338393)
Supplement: Attachment — Submitted filename: Response to Reviewers.docx [file pone.0338393.s002.docx]

**Response to the Reviewers Comment**

Journal: PLOS ONE

Manuscript Number: PONE-D-25-35010

Title: "**Magnitude of tuberculosis treatment outcomes and associated factors in public health institutions of Arba Minch town, Southern Ethiopia: A multi-centered retrospective study**”

Authors: Behailu Asmamaw, Temesgen Tamiru Tadesse, Rediet Yifru Mamo, Berhanu Amare Taye, Abrham Workineh Azale, Yohannes Chemere Wondmeneh, Dagmawi Nega Shibeshi, Eunice Borkor Bortequaye, Temesgen Teklu Ziza, Temesgen Esayas Bolliso, Tiwabwork Tekalign, Awoke Guadie^*^

**Dear Dr. Quinn,**

In response to your decision to revise our manuscript, we prepared the revised version of the manuscript according to the reviewers’ comments and suggestions. We have addressed the comments in the revised manuscript and in the response part. We show all changes we made in the response with yellow-highlight for deleted and red-highlight for corrected words, phrases or sentences. In the corrected manuscript titled “Manuscript”, only red-highlight was shown.

Thank you again for handling the manuscript.

Yours sincerely,

All the authors

**Editor Comment**

Dear Dr. Guadie,

Thank you for submitting your manuscript to PLOS ONE. After careful consideration, we feel that it has merit but does not fully meet PLOS ONE’s publication criteria as it currently stands. Therefore, we invite you to submit a revised version of the manuscript that addresses the points raised during the review process.

Please submit your revised manuscript by Oct 02 2025 11:59PM. If you will need significantly more time to complete your revisions, please reply to this message or contact the journal office at [plosone@plos.org](mailto:plosone@plos.org). When you're ready to submit your revision, log on to https://www.editorialmanager.com/pone/ and select the 'Submissions Needing Revision' folder to locate your manuscript file.

We look forward to receiving your revised manuscript.

Kind regards,

Frederick Quinn

Academic Editor

PLOS ONE

**Response:** Dear Editor, thank you very much for your effort to get our manuscript get appropriate reviewers on time. We also appreciate you giving us an extension (until October 30, 2025), which is crucial for us to compile our response according to reviewers comment.

**Comment 1:** Please ensure that your manuscript meets PLOS ONE's style requirements, including those for file naming. The PLOS ONE style templates can be found at.

<https://journals.plos.org/plosone/s/file?id=wjVg/PLOSOne_formatting_sample_main_body.pdf> and

<https://journals.plos.org/plosone/s/file?id=ba62/PLOSOne_formatting_sample_title_authors_affiliations.pdf>

**Response 1:** Thank you so much for the information. We have made sure that our revised manuscript follows the PLOS ONE requirements.

**Comment** **2**: In the online submission form, you indicated that [The data underlying the results presented in the study are available from the corresponding author with a reasonable request.]. All PLOS journals now require all data underlying the findings described in their manuscript to be freely available to other researchers, either 1. In a public repository, 2. Within the manuscript itself, or 3. Uploaded as supplementary information.

**Response 2:** Thank you so much for the information. Our data is included in the manuscript and raw data is available at the corresponding author with reasonable request.

**Comment** 3: PLOS requires an ORCID iD for the corresponding author in Editorial Manager on papers submitted after December 6th, 2016. Please ensure that you have an ORCID iD and that it is validated in Editorial Manager. To do this, go to ‘Update my Information’ (in the upper left-hand corner of the main menu), and click on the Fetch/Validate link next to the ORCID field. This will take you to the ORCID site and allow you to create a new iD or authenticate a pre-existing iD in Editorial Manager.

**Response 3:** Thank you so much for the information. The corresponding author’s ORCiD iD is available here: [**https://orcid.org/0000-0003-2753-4216**](https://orcid.org/0000-0003-2753-4216)

**Comment** **4**: Your ethics statement should only appear in the Methods section of your manuscript. If your ethics statement is written in any section besides the Methods, please move it to the Methods section and delete it from any other section. Please ensure that your ethics statement is included in your manuscript, as the ethics statement entered into the online submission form will not be published alongside your manuscript.

**Response**: Thank you so much for the concern. We have already put the ethical considerations at the end of the methodology (page 7, lines 201-205 in the revised manuscript).

**Ethical approval and consent to participants**

The ethical approval to conduct this study was obtained from the Arba Minch University	Ethical Review Board (IRB/23304/25), and a cooperation letter was given to the facilities. This	study had no contact with patients, and the information obtained regarding patients was made anonymous.

**Comment 5:** If the reviewer comments include a recommendation to cite specific previously published works, please review and evaluate these publications to determine whether they are relevant and should be cited. There is no requirement to cite these works unless the editor has indicated otherwise.

**Response:** thank you so much for the concern. In our case there is no any recommendation given by the reviewers to cite a specific article.

**Comment 6:** Please review your reference list to ensure that it is complete and correct. If you have cited papers that have been retracted, please include the rationale for doing so in the manuscript text, or remove these references and replace them with relevant current references. Any changes to the reference list should be mentioned in the rebuttal letter that accompanies your revised manuscript. If you need to cite a retracted article, indicate the article’s retracted status in the References list and also include a citation and full reference for the retraction notice.

**Response:** Thank you so much for the concern. We have ensured our reference list is complete and correct. In our case we have not used retracted articles as a reference.

**Reviewer #1 Comments**

Comment 1: The authors should work on describing the methodological procedures in more detail for the sake of reproducibility of this study. By clearly mentioning the inclusion criteria, sampling methods, how missing data were handled, and how potential biases were addressed.

**Response 1:** Thank you very much for your detailed comments. We greatly appreciate your suggestions regarding the need for more detailed methodological description to enhance the reproducibility of our study. Now, we have revised the methodology section to provide greater clarity and detail for reproducibility by including inclusion criteria, sampling methods, how missing data handled, and how potential biases addressed (Pages 4-6, lines 103-179).

**2.3. Eligibility criteria**

**2.3.1. Inclusion criteria**

The data of all patients diagnosed with TB and who were initiated on anti-TB treatment in public health institutions of Arba Minch town during the study period were included in the study. Patients who had full information in the unit TB register including clearly documented treatment outcomes, were considered eligible.

**2.3.2. Exclusion criteria**

Patients who were referred to other health facilities and/or had incomplete information were excluded from the study. Records were considered incomplete if they missed data on essential variables such as age, sex, or treatment outcomes (cured, treatment completed, treatment failure, loss to follow-up, death, or not evaluated).

The data of all patients diagnosed with TB and who were started on anti-TB treatment during the study period and had full information in the unit TB register were included in the study.

Patients who were referred and/or had incomplete information were excluded from the study. Patients’ data that lacked full information about age, sex, year of treatment, and treatment outcomes (cured, completed, treatment failure, loss to follow-up, death, and not evaluated) were considered incomplete data.

**2.4. Study design and sampling procedure**

This multi-centered, facility based retrospective study was conducted in public health facilities of Arba Minch town, Southern Ethiopia. Between September 2021 and August 2024, a total of 700,340 patients were seen across these public health institutions for various health services. Of these, 675 patients were diagnosed with all forms of tuberculosis (TB) and initiated on anti-TB treatment at the three facilities (Figure 1). Among them, 609 patients fulfilled the eligibility criteria and were included in the study (Figure 1). To maximize representativeness, eliminate sampling error, and enhance the reproducibility of the findings, the study employed a census of all eligible TB patients during the study period.

This multi-centered, facility-based retrospective study was conducted in public hospitals of Arba Minch town, Southern Ethiopia. The total registered patients in the public health institutions from September 2021 to August 2024 were 700,340. During the study period, a total of 675 TB patients were diagnosed as having all forms of TB and were linked to TB treatment clinics at the three facilities (Figure 1). All TB patients who started anti-TB treatment during the study period and had full information on TB treatment outcome records were included in the study. From all health facilities, study participants who fulfilled the inclusion criteria were selected by using a consecutive sampling technique, and a total of 609 TB patients who fulfilled the inclusion criteria were included in the study (Figure 1)

**2.7 Data quality control**

To minimize potential biases, a pre-test was conducted with 5% of the final sample size before the main data collection, and the checklist was revised based on the findings to ensure completeness and clarity. The pre-tested, structured questionnaire, prepared in English, was checked for consistency after the pretest.

A pre-test was conducted with 5% of the final sample size out of the study period before the actual data collection period. Based on the pre-test results, the checklist was modified as needed. The pre-tested structured questionnaire was prepared in English, and it was checked for completeness and consistency after the pre-test.

Comment 2: Please provide justifications for analytical approaches.

**Response 2:** We thank the reviewer for this important comment. In the revised manuscript, we have added justifications for our analytical approaches. Now, it is organized in line with the reviewer comment (Page 7, lines 185-199).

Data The collected for this study data were managed using EpiData version 3.1 software. The data were thoroughly checked for completeness, coded, and entered using EpiData version 3.1 into the software. The data was then exported and analyzed using SPSS version 26. Binary logistic regression was used to examine the association between the outcome variable and each independent variable. Variables with a p-value of < 0.25 in the bivariate analysis were considered potential candidates for multivariable logistic regression analysis. A variable with a p-value of ≤ 0.05 was considered statistically significant in its association with the outcome variable. Due to the dichotomous nature of the outcome variable (successful vs. unsuccessful treatment outcomes), logistic regression analysis was employed. This model was specifically chosen for its ability to estimate the odds of an event occurring while effectively adjusting for multiple covariates. Initially, a binary logistic regression analysis was performed to identify variables associated with the treatment outcome at a crude level. Any variables with a p-value of <0.25 in the binary analysis were considered a potential candidate for inclusion in the final (i.e., multivariable logistic regression) model to control for potential confounding and to identify independent predictors of treatment outcome. Statistical significance in the final model was established at a p-value of $\leq$0.05. Finally, model fitness was assessed using the Hosmer–Lemeshow goodness-of-fit test, and multicollinearity among independent variables was checked to ensure the reliability of the final regression model.

Comment 3: Please provide tools for analysis of predictors of mortality.

**Response 3:** We thank the reviewer’s comment on tools used for analysis of predictors of mortality. In our study, mortality is included as part of the composite outcome “unsuccessful treatment outcome,” which also comprises treatment failure, loss to follow-up, and not evaluated. As explained in **Response 2** **(Page 7, lines 185-199).** we used a multivariable logistic regression model to identify independent predictors of treatment outcome. This approach allows adjustment for potential confounding factors while examining associations between independent variables and the dichotomous outcome (successful vs. unsuccessful).

Comment 4: The findings listed in this research article mainly reiterate the known risk and known associations, and lacks in providing actionable new insights.

**Response 4:** We thank the reviewer for this insightful comment. While some of the findings in our study have been previously reported, our study provides important context-specific evidence for public health facilities in Arba Minch town. Notably, the overall treatment success rate (TSR) that we found in our study is 86.9%. This is below the target set by the World Health Organization end TB strategy, which aims to achieve a TSR of 90% by 2030. This indicates that additional efforts are required to improve TB treatment outcomes in this setting. The factors significantly associated with treatment success in our population (being unmarried, having extra-pulmonary TB, and being educated) highlight specific subgroups that may benefit from targeted interventions. For example, enhanced adherence support could be directed toward married patients or those with pulmonary TB, and health education campaigns could focus on populations with lower literacy levels. This could help reduce the burden of the disease by informing targeted interventions. Thus, although the identified predictors are consistent with known associations, our findings provide actionable insights for programmatic planning and local TB control efforts, emphasizing the need for tailored interventions to achieve WHO treatment success targets. Now, we considered the reviewer comment in the conclusion section (Pages 12-13, lines 361-369).

The study indicates a relatively positive rate of successful TB treatment outcomes (86.9%), even though it is below the WHO target of more than 90% TSR. Never married, attending formal education, and being diagnosed with EPTB were significantly associated with successful treatment outcomes. In this study, the overall TB treatment success rate was found to be 86.9%, which is significantly associations with being unmarried, attending formal education, and being diagnosed with EPTB. This finding is below the target set by the WHO end TB strategy, which aims to achieve a TSR of 90% by 2030. Therefore, This indicates that attention should be given to patients who are married, individuals with low educational levels, and patients diagnosed with pulmonary TB in order to improve the overall outcome of TB treatment in the Gamo zone. While the study benefits from being multi-centered and large sample size, future studies should focus on longitudinal studies to identify other potential socioeconomic and cultural factors that could affect the treatment outcomes of TB patients.

Comment 5: Discussion should be improved by addressing the global scenarios beyond Africa, including other countries like China, India.

**Response 5:** Thank you very much for this insightful comment. We strongly agree that including global scenarios is important, especially as China and India carry a major share of the global burden of TB, MDR-TB, and TB-HIV co-infection. We have revised the discussion with the addition of China, and India in the revised manuscript (Page 10-17, lines 285-506)

In addition, our treatment success rate is lower than that reported in Hunan Province, China (98.6%) [30] as well as the national average of 95% in 2022[1]. This difference may be attributed to China’s strong universal health coverage, substantial TB financing, and the consistent application of DOTS through its National TB Control Program. Similarly, in India 87.9% of TB treatment success rate were reported in 2023 [31], supported by its Revised National TB Control Program, which emphasizes early case detection, patient-centered treatment, and enhanced monitoring systems. (page 10, lines 285-291)

30. Gilmour B, Xu Z, Bai L, Alene KA, Clements ACA. Risk factors associated with unsuccessful tuberculosis treatment outcomes in Hunan province, China. Trop Med Int Health. 2022;27(3):290–9. <https://doi.org/10.1111/tmi.13720>

31. Prajapati AC, Shah T, Panchal S, Joshi B, Shringarpure K, Jakasania A, et al. Treatment outcomes and associated factors among patients with drug-sensitive tuberculosis on daily fixed-dose combination drugs: A cohort study from Ahmedabad, India. J Fam Med Prim Care. 2023;12(3):452–9. doi:10.4103/jfmpc.jfmpc_1331_22 (page 17, lines 500-506)

Comment 6: Addressing why HIV positivity, BMI are not a significant association.

**Response 6:** We thank the reviewer for this critical comment. In our study, HIV positivity and BMI were not significantly associated with TB treatment outcomes. We have now revised the discussion to explain this lack of association in the revised manuscript (Pages 11-12, lines 332-349).

.

While HIV positivity, advanced age, being male, and malnutrition are mentioned as the most common factors associated with unfavorable treatment outcomes, our study did not demonstrate any significant association of outcomes with these factors. Although many studies [1, 8, 27] stated that HIV positive, advanced age, male sex, and malnutrition are commonly associated with unfavorable TB treatment outcomes, our study did not find significant associations with these factors. The finding that HIV positive status was not statistically associated with poor TB treatment outcomes in our study, contrary to global trends, likely originates from the effectiveness of the local integrated TB-HIV collaborative services. The most probable primary reason is the high (98.5%) ART coverage and timely initiation (Table 2) among TB-HIV co-infected patients. Antiretroviral therapy rapidly suppresses the HIV virus, leading to immune reconstitution, which allows the patient's body to respond effectively to the anti-TB drugs, mitigating the risk of death and treatment failure typically seen in immunocompromised individuals [1].

The prominent consumption of low-fat staple foods in the study area like *Enset* (including its products: *Kocho*, *Bulla*, and *Godere*), may directly contribute to lower population BMI values. Since BMI is a simple measure that does not differentiate between lean muscle mass and fat stores, a low BMI in this context may primarily reflect limited dietary fat intake rather than severe protein-energy malnutrition. Consequently, BMI may be an inadequate indicator of specific nutritional deficits (e.g., in essential fatty acids or fat-soluble vitamins) crucial for immune function and recovery. This limitation likely accounts for the observed lack of association between BMI and the measured TB treatment outcomes in this population.

**Reviewer #2 Comments**

Comment 1: The manuscript investigated the outcome of tuberculosis treatment and associated factors in multicenter facilities. The research being done in multicenter make the results more representative of broader population. The study is clearly presented and the methodology meets the PlOS One guidelines. The manuscript is technically sound, and the data supports the conclusions. **Overall impression:** the study addresses an interesting research question. Having said these, few minor issues must be addressed before publication.

Response 1: We sincerely thank reviewer 2 for the encouraging and constructive feedback. We are pleased that the reviewer recognized the multicenter design, which enhances the representativeness of our findings for the broader population. We are also pleased that the reviewer found the study clearly presented in line with PLOS ONE guidelines.

Comment 2: In the title of the study: “cross-sectional study” must be included in the title

**Response 2:** We thank the reviewer for this valuable suggestion. In response, we have revised the title of the manuscript to explicitly include the study design. Now, it is corrected in the revised manuscript (Page 1, lines 1-3).

Magnitude of tuberculosis treatment outcomes and associated factors in public health institutions of Arba Minch town, Southern Ethiopia: A multi-centered retrospective cross-sectional study

Comment 3: In the abstract: In the abstract part (line 31 to 33) and the on results (line 226 and 227); based on your results and table 3, educational status was not statistically significant. And the variables which are associated with successful treatment outcome should be written as “being unmarried” and “extra-pulmonary TB” instead of marital status and type of TB.

**Response 3:** We sincerely thank the reviewer for the careful reviewing the abstract and Result section which we made typographic error (for educational status) and ordering problems when mentioned in the abstract section. Now, we carefully go through the abstract and result section as well as the reviewer’s suggested word explanation corrected in the revised manuscript (Page 2, line 32-33).

In the multivariate logistic regression, marital status being unmarried (p=0.023), educational level (p=0.028), and the type of TB extra-pulmonary TB (p=0.017) have been found significantly associated with successful treatment outcome.

Comment 4: In the introduction part: Write the reference of line 52 to 54.

**Response 4:** Thank you for your valuable feedback. We have added the following reference to support the statements in lines 52-54 of the introduction part manuscript (Page 2, line 55 and page 14, lines 417-418).

4.	Guidelines for Clinical and Programmatic Management of TB, TB/HIV, DR-TB and Leprosy in Ethiopia.

Comment 5: In the methodology part: Language and grammar, line 128 to 130 is not written as a sentence, please rewrite it.

**Response** **5:** Thank you so much for the comment. Now, the reviewer’s comment has been considered and corrected in the revised manuscript (Page 5, lines 130-134).

The study also collected many Cclinical characteristics such as the type of TB, TB registration category, GeneXpert *Mycobacterium tuberculosis*/rifampicin resistance (MTB/RIF) assay diagnosis results, HIV status, initiation of antiretroviral therapy (ART), and indications of presumptive drug resistance (PDR). During data collection data were collected through experienced staff working in the TB treatment units were involved.

Comment 6: Line 224, the subtitle should be written as “factors associated with successful tuberculosis treatment outcome” since the study is assessing the associated factors of successful treatment outcome instead of factors associated with tuberculosis treatment outcome.

**Response 6:** Thank you so much for the comment. In order to accurately reflect that our analysis focused on factors linked to successful treatment outcomes, we have added the word “successful” to the existing subtitle in the revised manuscript (Page 8, line 236).

**3.4. Factors associated with successful TB treatment outcome**

Comment 7: Line 226 and 227; education is not significant from your table (table 3). The texts “marital status” and “type of TB” should be written as being unmarried, and having extra-pulmonary TB as they are the ones which was associated with successful treatment outcome. In addition, use past tense when you talk about your results.

**Response 7:** We sincerely appreciate the reviewer’s careful and critical observation, which identified a mismatch between the manuscript text and Table 3. We confirm that the value of p=0.088 in Table 3 was indeed a typographic error and has been corrected to the accurate value of p=0.028 (as it is seen in the abstract, result, and discussion). Furthermore “Marital status” is replaced by “being unmarried” and “type of TB” is replaced by “having extra-pulmonary TB” as suggested by the reviewer. To make the writing consistent, we also replaced “ever married” by “Married” in Table 1, and 3 as shown in the revised manuscript (Pages 2-9, lines 32-244)

In the multivariate logistic regression, marital status being unmarried (p=0.023), educational level (p=0.028), and the type of TB having extra-pulmonary TB (p=0.017) have been found significantly associated with successful treatment outcome. (Page 2, lines 32-34)

These are educational status (p=0.028), marital status being unmarried (p=0.023), and type of TB having extra-pulmonary TB (p=0.017) (Table 3).

Those study participant who had formal education were 2 times more likely to have successful treatment outcome (AOR= 1.67; 95% CI=1.05-2.66; p=0.028) than their counterparts. Those participants who were never unmarried during the time of treatment were 2 times more likely to have successful treatment outcome (AOR= 1.64; 95% CI=1.05-2.47; p=0.023) than ever-married participants and participants with EPTB were almost 4 times more likely to have successful treatment outcome (AOR=3.8; 95% CI=1.23-11.7; p=0.017) than participants with pulmonary TB. (Pages 8-9, lines 239-248)

**Table 1.** Socio-demographic characteristics of study participants (page 19, lines 533-534)

| Variables | Characteristics | Frequency | % |
| --- | --- | --- | --- |
| Age (year) | <20 | 129 | 21.2 |
|  | 21-40 | 327 | 53.7 |
|  | 41-60 | 111 | 18.2 |
|  | >60 | 42 | 6.9 |
| Gender | Male | 380 | 62.4 |
|  | Female | 229 | 37.6 |
| Marital status | Never unmarried | 281 | 46.1 |
|  | Ever Mmarried | 328 | 53.9 |
| Educational level | Informal education | 136 | 22.3 |
|  | Formal education | 473 | 77.7 |
| Body mass index | Under weight | 326 | 54.6 |
|  | Normal weight | 251 | 42.1 |
|  | Overweight | 19 | 3.1 |

**Table 3.** Factors associated with tuberculosis treatment outcome in public health institutions of Arba Minch town (n=609) (page 21, lines 551-552).

| Variables | Category | TB-TO | | COR (95% CI)^a^ | P-value | AOR (95% CI)^b^ |
| --- | --- | --- | --- | --- | --- | --- |
|  |  | Successful | Unsuccessful |  |  |  |
| Educational Status | Informal education | 100 | 36 | 1 |  | 1 |
|  | Formal education | 380 | 93 | 0.68(0.43-1.05) | 0.0828 | 1.67(1.05-2.66) *** |
| Marital status | Never Unmarried | 210 | 71 | 1.57(1.06- 2.32) | 0.023 | 1.64(1.05-2.47) *** |
|  | Ever-mMarried | 270 | 58 | 1 |  | 1 |
| Alcohol history | Yes | 237 | 55 | 1.31(0.88-1.94) | 0.174 | 1.08(0.70-1.67) |
|  | No | 243 | 74 | 1 |  | 1 |
| Smoking history | Yes | 361 | 105 | 1 |  | 1 |
|  | No | 119 | 24 | 0.69(0.42-1.13) | 0.143 | 1.40(0.81-2.42) |
| Type of TB | Pulmonary | 335 | 102 | 1 |  | 1 |
|  | Extra pulmonary | 135 | 21 | 3.85(1.26-11.77) | 0.017 | 3.80(1.23-11.7) *** |
|  | Unknown | 10 | 6 | 1.97(0.69-5.55) | 0.199 | 2.00(0.69-5.74) |
| HIV-Coin | Yes | 113 | 20 | 1.67(0.99-2.82) | 0.052 | 0.62(0.36-1.06) |
|  | No | 367 | 109 | 1 |  | 1 |
| ART-Init | Yes | 111 | 20 | 1.63(0.97-2.76) |  | 1.89(0.14-24.5) |
|  | No | 369 | 109 | 1 |  | 1 |

TB-TO=TB treatment outcome, COR=Crude Odd Ratio, AOR=Adjusted Odds Ratio, HIV-Coin=Human Immunodeficiency Virus Co-infection, ART-Init= Antiretroviral initiation, ***=p< 0.05. Values in a column indicated in letters “a” and “b” show the bivariate and multivariate logistic regression results respectively.

**Table 4.** Comparing TB treatment outcome and associated factors in prior studies (page 22, lines 555-556)

| Study area | Sample size (number) | Methodology | TSR (%) | Associated factors | Reference |
| --- | --- | --- | --- | --- | --- |
| Arba Minch, Ethiopia | 609 | Retrospective | 86.9 | Formal education, never unmarried, and EPTB (+) | This study |
| Gondar, Ethiopia | 1584 | Retrospective cross-sectional | 60.1 | Age 30-39.9, smear negative, and being EPTB, retreatment, HIV positive (-) | [26] |
| Woldia, Ethiopia | 270 | Cross-sectional | 80.7 | Older age, female sex, year of registration in 2015, and being HIV positive (-) | [27] |
| Afar, Ethiopia | 380 | Retrospective | 81.8 | Older age, HIV Reactive status (-); while (four-week attendance, sputum follow-up test, and being female (+) | [24] |
| Wolaita, Ethiopia | 232 | Retro-cohort | 82.5 | No statistically significant associated factor | [10] |
| Gibe, Ethiopia | 400 | Cross-sectional | 85.5 | Age greater than 45, male sex, travel distance over 10 km, lack of family support, bedridden functional status (-) | [12] |
| Debretabor, Ethiopia | 303 | Retrospective | 87.1 | Female sex, rural residence, smear negative at 2^nd^ month of treatment (-) | [20] |
| Jimma, Ethiopia | 1249 | Retrospective | 88.2 | Smear-negative, Extra-pulmonary TB, unknown HIV status (-) | [28] |
| Harare, Ethiopia | 1236 | Retrospective | 92.5 | Female, HIV negative, weight 20-29 kg (+); while Age > 54 (-) | [8] |
| Enfranz, Ethiopia | 417 | Retrospective | 94.8 | No statistically significant associated factor | [29] |
| Easter Cape, South Africa | 427 | Ambidirectional | 65.8 | HIV co-infection, history of smoking (-) | [22] |
| Ashanti region, Ghana | 891 | Retrospective | 68.4 | Retreatment (-); while Age less than 20 or age range b/n 51-60, pre-treatment weight > 35kg (+) | [20] |
| Anambra & Oyo, Nigeria | 1281 | Cross-sectional | 75.8 | Geographical location, facility tier/type (-) | [23] |
| Mogadishu, Somalia | 385 | Cross-sectional | 81.5 | Married, educated, HIV-negative, new treatment, knowledge of TB (+) | [24] |
| Hunan province, China | 308,860 | Retro-cohort | 98.6 | Age, male, being severally ill, TB treatment history, year of diagnosis (-) | [30] |
| Ahmedabad, India | 8301 | Retro-cohort | 87.9 | Age 45-54 (-) | [31] |
| Ethiopia* | NS | Survey report | 86.0 | Age, gender, HIV, under-nutrition, diabetes mellitus, alcohol, drug resistance, smoking, poverty (-) | [1] |
| Africa* | NS | Survey report | 88.0 | Age, gender, HIV, under-nutrition, diabetes mellitus, alcohol, drug resistance, smoking, poverty (-) | [1] |
| World* | NS | Survey report | 88.0 | Age, gender, HIV, under-nutrition, diabetes mellitus, alcohol, drug resistance, smoking, poverty (-) | [1] |

(+)= Significantly associated with successful treatment outcomes, (-)=Significantly associated with unfavorable treatment outcomes, EPTB= Extra-pulmonary tuberculosis, TSR:=Treatment success rate, Retro-cohort=Retrospective cohort, *=Average report in 2024, NS=Not stated, Prosp-cohort=Prospective cohort

Comment 8: Being never married will be difficult to find from medical records it would be better if it is written as unmarried during the time of treatment.

**Response 8:** We thank the reviewer for this suggestion. As recommended, we have replaced “being never married” with “unmarried during the time of treatment” and to make the text align with the previous recommendations, we replaced “ever married” with “married” throughout the manuscript (Page 9, lines 243-248).

Those participants who were never unmarried during the time of treatment were 2 times more likely to have successful treatment outcome (AOR= 1.64; 95% CI=1.05-2.47; p=0.017) than ever-married participants and participants with EPTB were almost 4 times more likely to have successful treatment outcome (AOR=3.8; 95% CI=1.23-11.7; p=0.020) than participants with pulmonary TB.

Comment 9: On discussion part; when you discuss researches done in Ethiopia, please mention Ethiopia in addition to the name of the place so that the international readers will know it is Ethiopia.

**Response 9:** We thank the reviewer for this helpful suggestion. Now, we have revised the discussion part carefully including “Ethiopia” and other countries location in the revised manuscript (Pages 9-10, line 255-291).

The magnitude of successful treatment outcomes among TB patients was found to be 86.9%, which is a bit lower than the world (88%) and African (88%) average TB treatment success rate for new and relapse cases reported in 2024 [1]. However, tThis result is nearly similar to studies conducted in Gibe Woreda, Southern Ethiopia (85.5%)[12] and Debre Tabor Town, Northwestern Ethiopia (87.1%)[20]. Such consistency in outcomes could suggest comparable healthcare practices or disease management strategies in these regions (Table 4).

In contrast to our finding, other studies from various locations have reported significantly lower TB treatment success rates in Ethiopia and other countries in Africa. For example, a study in Ghana (five directly observed therapy short-course (DOTS) centres at the Atwima Nwabiagya district) found a lower TB treatment success rate (68.5%)[21], while in South Africa (Eastern Cape hospitals) recorded a mere an even lower (65.8%) rate of treatment success [22]. SimilarlyMoreover, studies in two Nigeria states (Anambra and Oyo) and Somalia (across seven public TB management units in Mogadishu) demonstrated a lower TB treatment success rate of 75.8%[23] and 81.8%[24], respectively. Lower TB treatment success rates were also observed across various regions in Ethiopia, highlighting significant internal differences that may reflect localized challenges in healthcare delivery. Further disparities were observed in other parts of Ethiopia including Specifically, TB treatment success rates reported in the Afar region (81.8%)[25], Gonder (60.1%)[26], Wolayta (82.5%)[10], and Woldia (80.7%)[27] (Table 4). These regional variations may be attributed to several factors, including differences in study periods and sample sizes. The discrepancies also highlight how regional healthcare systems and patient demographics can ultimately influence treatment outcomes. adherence and treatment completion. A comprehensive understanding of these discrepancies is essential for designing effective TB control strategies across the country.

On the other hand, thise current study's TB treatment success rate was lower than those reported in Harar, Eastern Ethiopia (92.5%)[8], Jimma, Southwestern Ethiopia (88.3%)[28], and Enfraz Health Center, Northwestern Ethiopia (94.8%)[29] (Table 4). The higher success rates in these studies may be attributed to different methodologies, particularly the use of larger sample sizes, which typically enhance the reliability of findings. Additionally Moreover, the types of healthcare facilities and their level may significantly influence treatment outcomes. Factors such as the demographic characteristics of the patient population and the degree of urbanization in the settings could also play a crucial role in affecting these outcomes. In addition, our treatment success rate is lower than that reported in Hunan Province, China (98.6%) [30] as well as the national average of 95% in 2022[1]. This difference may be attributed to China’s strong universal health coverage, substantial TB financing, and the consistent application of DOTS through its National TB Control Program. Similarly, in India 87.9% of TB treatment success rate were reported in 2023 [31], supported by its Revised National TB Control Program, which emphasizes early case detection, patient-centered treatment, and enhanced monitoring systems.

Comment 10: On discussion part on line 271 to 274 you wrote “married individuals in Arba Minch area share multiple competing sociocultural obligations, such as workloads to maintain family incomes and family responsibilities that could detract them from their health management efforts, unlike never-married individuals who may prioritize their health more effectively”. How do you reach to that conclusion

**Response 10:** We thank the reviewer for this insightful comment. We acknowledge that our study did not directly measure adherence differences by marital status. To address this, we have revised the discussion section, presenting the statement as a plausible explanation based on observations and findings from previous studies, rather than a definitive conclusion. The improved discussion can be found in the revised manuscript (Pages 10-17, lines 292-514).

With analysis of Mmultivariate logistic regression model analysis showed that, participants who had never been married were unmarried during the time of treatment had 64% higher odds of having a successful treatment outcome (AOR = 1.64; 95% CI = 1.05-2.47; p=0.017) compared to those who were ever married. This finding contrasts with previous studies, which demonstrated unmarried individuals had a lower social support system[10,24,27]. One possible explanation for this difference is that married individuals in Arba Minch area share multiple competing sociocultural obligations, such as workloads to maintain family incomes and family responsibilities that could detract them from their health management efforts, unlike never-married individuals who may prioritize their health more effectively. They may have more time and resources to dedicate to their health and exhibit higher levels of attention toward health behaviors. Contrary to literature emphasizing marital support, our findings suggest that in the Gamo zone, marriage may introduce barriers such as treatment interruptions and concealment of their symptoms due to the stigma of the disease and fear of losing a partner, which could lead to late presentation and make the outcomes poor. The most likely explanation could be due to married individuals in the Gamo zone may face competing family and household responsibilities, such as caregiving, income-generating activities, and maintaining household obligations, which could interfere with adherence and clinic attendance. Indeed, other studies in Southern Ethiopia found that being married was associated with lower odds of successful TB treatment outcome [32]. A study conducted within Gamo Gofa zone (a similar study area for our study) also highlighted that family dynamics, such as a patient's failure to disclose their TB status to household members, were strongly associated with non-adherence [33]. Based on our finding and these evidences [32, 33], the burden of marital and household commitments are critical contributing factors to suboptimal treatment success in this specific context, suggesting the vital need for future research to deeply explore and mitigate these underlying mechanisms. Our study is deficient in addressing how factors such as socioeconomic status, cultural norms and stigma, and household dynamics affect the treatment outcome among married patients. Future studies should explore a longitudinal or mixed-methods design to explore how the interplay of those factors and marital status affect the treatment of TB (pages 10-11, lines 292-310)

32.	Agazhu HW, Assefa ZM, Beshir MT, Tadesse H, Mengstie AS. Treatment outcomes and associated factors among tuberculosis patients attending Gurage Zone Public Hospital, Southern Nations, Nationalities, and People’s Region, Ethiopia: an institution-based cross-sectional study. Front Med. 2023;10:1105911.

33.	Ajema D, Shibru T, Endalew T, Gebeyehu S. Level of and associated factors for non-adherence to anti-tuberculosis treatment among tuberculosis patients in Gamo Gofa zone, southern Ethiopia: cross-sectional study. BMC Public Health. 2020;20(1):1705. (page 17, lines 507-514).
